# Fine Mapping and Candidate Gene Prediction of a Pleiotropic Quantitative Trait Locus for Yield-Related Trait in *Zea mays*

**DOI:** 10.1371/journal.pone.0049836

**Published:** 2012-11-21

**Authors:** Ruixiang Liu, Haitao Jia, Xiaoliang Cao, Jun Huang, Feng Li, Yongsheng Tao, Fazhan Qiu, Yonglian Zheng, Zuxin Zhang

**Affiliations:** 1 National Key Laboratory of Crop Genetic Improvement, Huazhong Agricultural University, Wuhan, China; 2 College of Agronomy, Hebei Agricultural University, Baoding, China; New Mexico State University, United States of America

## Abstract

The yield of maize grain is a highly complex quantitative trait that is controlled by multiple quantitative trait loci (QTLs) with small effects, and is frequently influenced by multiple genetic and environmental factors. Thus, it is challenging to clone a QTL for grain yield in the maize genome. Previously, we identified a major QTL, *qKNPR6*, for kernel number per row (KNPR) across multiple environments, and developed two nearly isogenic lines, SL57-6 and Ye478, which differ only in the allelic constitution at the short segment harboring the QTL. Recently, *qKNPR6* was re-evaluated in segregating populations derived from SL57-6×Ye478, and was narrowed down to a 2.8 cM interval, which explained 56.3% of the phenotypic variance of KNPR in 201 F_2∶3_ families. The QTL simultaneously affected ear length, kernel weight and grain yield. Furthermore, a large F_2_ population with more than 12,800 plants, 191 recombinant chromosomes and 10 overlapping recombinant lines placed *qKNPR6* into a 0.91 cM interval corresponding to 198Kb of the B73 reference genome. In this region, six genes with expressed sequence tag (EST) evidence were annotated. The expression pattern and DNA diversity of the six genes were assayed in Ye478 and SL57-6. The possible candidate gene and the pathway involved in inflorescence development were discussed.

## Introduction

Maize grain yield (GY) is the most important breeding goal, and is one of the most complex traits [1], because it comprises several yield components including kernel number (KN) and kernel weight (KW). Furthermore, the KN per ear measurement can be further classified into kernel row number (KRN) and kernel number per row (KNPR). Previous studies suggested that yield components always show higher heritability than GY [1]–[2], and selection of certain yield components could be more effective than direct selection for GY itself [3]–[4]. Thus, geneticists and breeders have strived to understand the genetic basis underlying maize GY, and its components, by quantitative trait locus (QTL) mapping approaches. Numerous QTLs for GY-related traits have been identified on maize chromosomes [5]. Those identified chromosome regions also provide targets for QTL pyramiding and gene cloning.

Our current knowledge of the molecular regulation and genetic basis of GY mainly comes from cloned mutant genes that are involved in the regulation of inflorescence architecture and development [6]. Typically, three *ramosa* genes, *ra1* on 7.02bin, *ra2* on 3.03bin and *ra3* on 7.04bin, separately encode a C2H2 Zinc-finger protein [7], a LOB domain protein [8] and a Trehalose phosphatase [9]. Mutation of each of the three genes results in the spikelet-pair meristems (SPMs) at the base of inflorescence to transition into branch meristems (BMs), leading to branched ears and tassels with increased degrees of branching. The second type of cloned genes is associated with meristem initiation and maintenance. *Barren stalk1 (ba1*) encodes a non-canonical bHLH (basic helix-loop-helix) domain protein [10] that regulates the initiation of all axillary meristems. Mutations in *ba1* result in mutant plants lacking vegetative tillers, ear tassel branches and spikelets [11]. *Barren inflorescence2* (*bif2*) encodes a serine/threonine protein kinase that regulates polar transport of auxin [12]. *Bif2* mutants produce rudimentary ears and tassels that occasionally produce spikelets [13]. Moreover, *thick tassel dwarf1* (*td1*) and *fascinated ear2* (*fea2*) encode two homologs of Arabidopsis CLAVATA proteins [14]–[15]. Mutation of either of the two genes influences inflorescence development by affecting the size or maintenance of the inflorescence meristem (IM). Maize GY is highly associated with female inflorescence development. The cloning of mutant genes involved in the regulation of ear architecture and development is undoubtedly helpful to understand the developmental regulation of GY and its components, but the genetic basis of the quantitative variation of GY and its components remains unknown.

Numerous QTLs and/or QTL clusters for GY-related traits have been identified in diverse populations; however, the cloning of QTLs remains difficult in the maize genome because of its large size and highly repetitive sequence [16]. Among identified QTLs, a clustered QTL for GY and its components on 6.01–6.03bin of the maize genome was repeatedly detected in previous studies. Ajmone-Marsan et al. (1995) reported a clustered QTL for GY in *umc59a-umc21* of 6.02–6.03bin that explained 24.5% of the phenotypic variance [17]. Frascaroli et al. (2009) detected a QTL for GY and KN in *phi075-bnlg1371* (6.01bin) in two testcross populations [18]. Peng et al. (2011) also found a pleiotropic QTL simultaneously controlling GY and KN on 6.02–6.03bin across multiple environments [19]. Moreover, many QTLs for GY-related traits were identified on 6S of the maize chromosome by multiple groups, such as a QTL for ear length (EL) in *phi126-bnlg1371* [20], *bnlg1538-bnlg391* [21] and *phi126-npi235* [1]. Liu et al. (2011) identified a QTL for KNPR around *phi031* in *Y1-umc1257* (6.01–6.02bin) [22], and Tan et al. (2011) identified a QTL for kernel weight in *umc1656-umc1796* (6.02–6.04bin) [23]. Our group identified a chromosome segment around *gpc2* harboring a QTL for KNPR across four environments, designated as *qKNPR6* [24].

**Figure 1:**
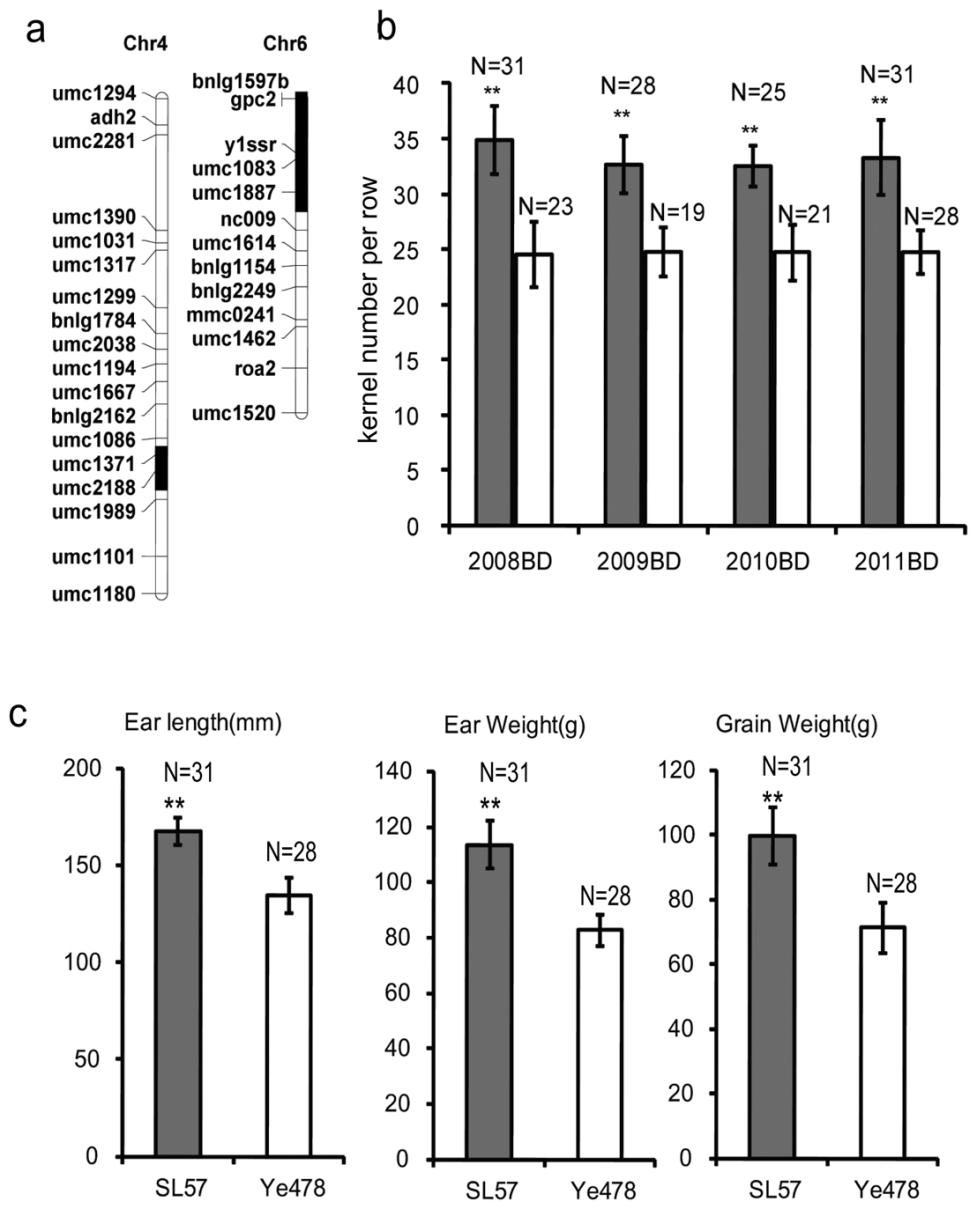
Distribution of the introgressed segments and phenotype of SL57. 1a: Distribution of the introgressed segments on maize chromosome 4 and 6 in SL57. The introgressed segments were identified by SSR screening. The white box represents the chromosome with the same genotype as Ye478, and the black box represents the introgressed segment. The genetic similarity between Ye478 and SL57 is 97.6%. 1b: The kernel number per row of SL57 and Ye478 under four environments. The white bar represents Ye478, and the black bar represents SL57. The horizontal axis shows four environments: Baoding in 2008 (2008BD), Baoding in 2009 (2009BD), Baoding in 2010 (2010BD) and Baoding in 2011 (2011BD). The vertical-axis shows the kernel number per row. N: The number of samples. 1c: The ear length, ear weight and grain weight of SL57 and Ye478 detected in 2010 at Baoding (northern China).

**Table 1:** Primers for QTL mapping and gene specific primers for real-time PCR.

	Marker/gene	Primer_F	Primer_R
Primers for QTL mapping	N6M19	TAGGTAGGCTACTAGGCTAA	AAGAAGAACATAAATTGGTACG
	N6D139	GGGTGAAGTGTGGAGAGA	GCTTTCTACAGGGATGTGT
	N6M30	GCATTGTTTGACTGGACTAG	AAGAGATAGAGCAGGACACT
	N6D262	CGGCAACCGATTAGTTAT	CGTCTGGGAAGAGAGATG
	N6D354	CTGCCCGCACAATCATTT	AGGAGAGTGAGGTGAGAAG
	N6D389	CGCTTGAAATGGAAAGGTAG	CTGCTGCTGGTCTACAAC
	N6M46	TGAACAGTGTGCTAGAGTG	TGAATTGCCAGTTGAATGC
	N6D543	GGTCCAGGTCCAATGAAC	ATTACGCACGCAATTAGC
	N6M64	TCATCACCAACCCTTCCA	ATCAGCAGGTCGTCGTAT
	N6M66	TGATGCGACACTGATTAGAT	CTTTGCGATGTCCTCCTATA
	N6M123	GAATCCTTGAGACCTTGACA	CCACCAGACGATGATGAAT
	N6M137	CGAGAACGGAAGTAGTACC	TCATTGCTGTCCAAATTGAC
Primers for real-time PCR	GRMZM2G128485	GGAAGGCTACGAGTACAGAAG	CAGGCACGAGGTCAACAC
	GRMZM2G428518	CGACCTCCGCTACTCCATC	GTTGTGTTGCCGCCGAAG
	GRMZM2G128574	AGGCTGGTATTGACTGATG	GGACACGAAGGTTCTCTG
	GRMZM2G119678	AGTAGGAGGATGGAGGATTG	CGCTGAATGGTTGTTGTTG
	GRMZM2G119714	TGGAGAACTCGGGTAAAT	CTGCGACTCTTGTGCTAA
	GRMZM2G128560	TCGGCTTCTTCAAGGACAGGA	CAAACGGGAGCGTCAAATCA
	Actin (GRMZM2G030169)	GACGCAGAGGACATTCAG	GGCTTCATCACCAACATAAG

These results highlight the importance of 6.01–6.03 bin for determining GY and its components, as well as providing a key target for QTL cloning. An optional strategy to finely map and clone a QTL is to create a large segregating population by crossing nearly isogenic lines (NILs) that differ only in the allelic constitution at the short chromosome segment harboring the target QTL (QTL-NILs). In such a population, because of the absence of other segregating QTLs, the target QTL becomes the major source of genetic variation, i.e., the QTL is considered Mendelized [25]. Thus, QTL detection power, resolution, and genetic effect can be significantly improved [16]. Previous studies in our laboratory confirmed a line, SL57, containing an introgressed segment flanked by *gpc2* and *umc1857*, which showed higher KNPR, longer ears and higher GY than the recurrent parent Ye478, an elite inbred in the Reid heterotic group. This implied that the introgressed segment encodes a pleiotropic gene, or several linked genes, which could affect the performance of multiple traits. In the present study, we used two inbred lines, SL57 and Ye478, to develop a new mapping population. We then combined linkage mapping and substitution mapping strategies to: 1) re-evaluate *qKNPR6* and its genetic effect underlying the introgressed segment in SL57; 2) fine map *qKNPR6* to a <1 cM interval; and 3) infer potential candidate genes responsible for *qKNPR6*.

**Figure 2:**
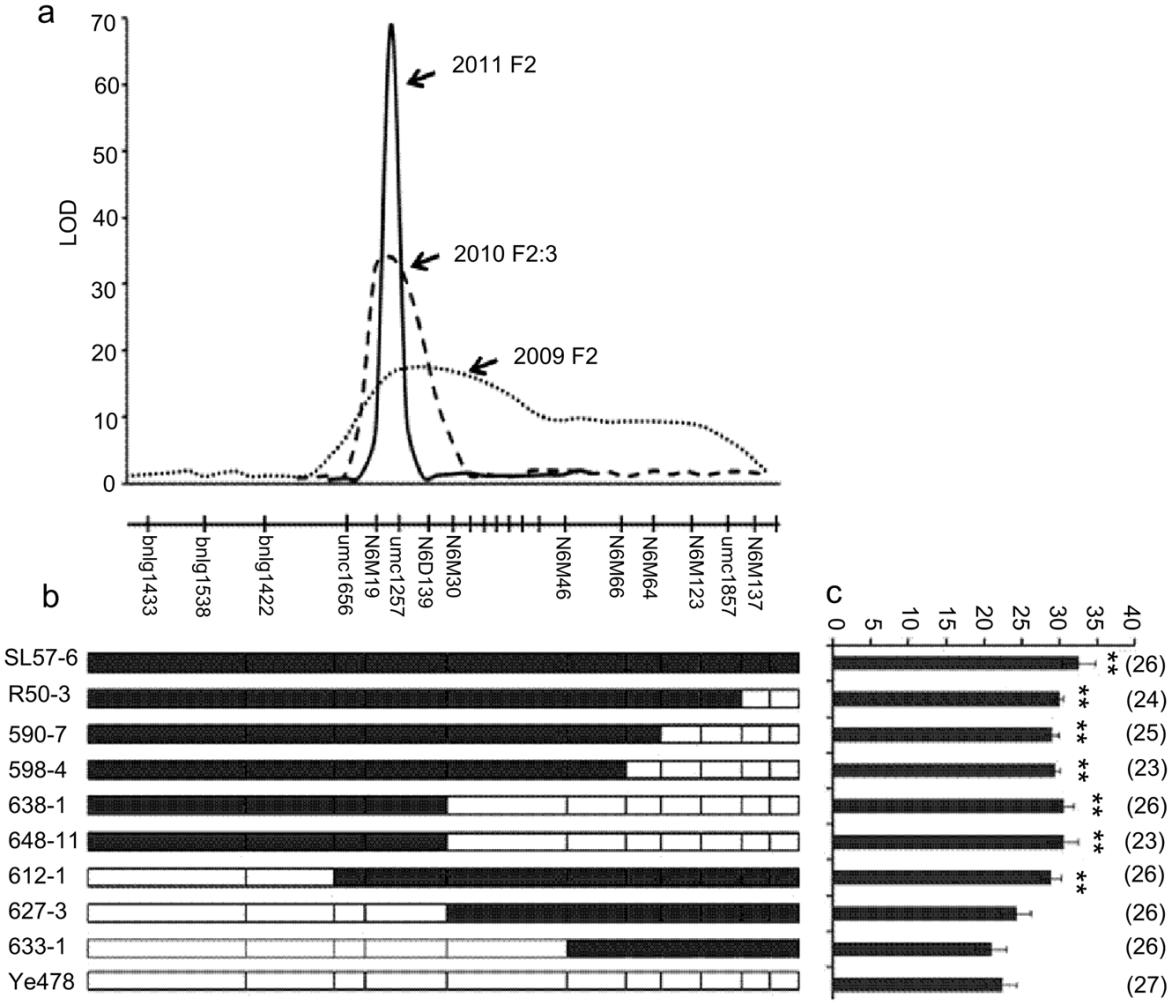
QTL interval for kernel number per row identified in different populations. 2a: The QTL interval detected using different segregation populations; the high LOD score supported the QTL interval. 2b: The genotypes of sub-NILs derived from the recombinant chromosomes. The black boxes or bars represent marker genotypes of SL57-6, the white boxes represent marker genotypes of Ye478. 2c: The phenotypes of sub-NILs detected in summer 2010 at Baoding (northern China). The amount in parentheses showed the number of samples. **: Significant difference at P = 0.01.

**Table 2:** Phenotypic effects of introgression segments in chromosome 4 and 6 detected in three environments.

NIL	Chromosome 6					Chromosome 4			Kernel number per row					
	bnlg1433	bnlg1538	bnlg1422	umc1656	umc1857	umc2188	umc1371	umc1999	2010BD		2010SY		2011BD	
									Mean±SD	P value	Mean±SD	P value	Mean ±SD	P value
SL57	A	A	A	A	A	A	A	A	31.1±1.3	1.10E-05	29.5±1.5	5.30E-03	33.8±1.9	5.20E-17
SL57-6	A	A	A	A	A	B	B	B	30.1±1.7	2.00E-04	29.2±1.3	6.20E-03	31.3±2.3	1.10E-11
SL57-4	B	B	B	B	B	A	A	A	25.3±0.8	0.18	24.5±2.7	0.5	25.5±1.3	0.06
Ye478	B	B	B	B	B	B	B	B	24.4±1.1		23.3±2.2		24.7±0.8	

A and B represent marker genotypes that are the same as SL57 and Ye478, respectively.

2010BD, 2010SY and 2011BD indicate that kernel number per row was measured in 2010 at Baoding (Northern China), in 2010 at Sanya (Southern China) and in 2011 at Baoding, respectively.

## Materials and Methods

### Plant Materials

The SL57 is a chromosome segment substitution line that contains two chromosome segments, on chromosomes 4 and 6, introgressed in the Ye478 genetic background, as revealed by 173 simple sequence repeat (SSR) markers (Figure 1a). The GY-related traits: KNPR, EL, ear weight (EW) and grain weight (GW) in SL57 are significantly higher than in Ye478 (Figure 1b and 1c). A QTL for KNPR (*qKNPR6*) was identified across four environments within the introgressed segment on chromosome 6 [24]. SL57 and Ye478 were crossed to separate the introgression segment on chromosome 4 from the one on chromosome 6 in SL57, which created two new lines: SL57-4 containing an introgressed segment on chromosome 4, and SL57-6 containing single introgressed segment on chromosome 6. SL57-6 displayed a similar phenotype in terms of GY-related traits to that of SL57, but is genetically more similar to Ye478 (Figure 1); thus, SL57-6 was defined as a QTL-NIL of Ye478.

Subsequently, Ye478 and SL57-6 were crossed to develop a QTL mapping population. Approximately 600 SL57-6×Ye478 F_2_ plants were grown during the summer of 2009, of which 193 individuals were open-pollinated for linkage map construction and QTL mapping, 201 individuals were self-pollinated to develop F_2∶3_ families for remapping of the target QTL, and the remaining individuals were genotyped to detect the recombinants that were then self-pollinated to develop homozygote recombinant lines. The phenotypes of the F_2∶3_ families and homozygote recombinant lines were evaluated by a field experiment using a randomized complete block with three replications, with twenty-two individuals per block, in summer 2010 at Baoding (Northern China). 12,800 F_2_ plants were grown in summer 2011 to finely map the QTL. 1000 randomly selected F_2_ plants were genotyped and used for QTL mapping. Using PCR-based markers within *qKNPR6*, 363 recombinants were selected. Of these recombinants, 191 plants carrying recombinant chromosomes were open-pollinated to evaluate the phenotype. The other recombinants were selfed to develop homozygous lines with overlapping in the introgression segment (hereafter termed sub-NIL). Ten sub-NILs, Ye478 and SL57-6 were grown at Sanya (Southern China) in winter 2011 with three replications, with twelve individuals per block. The GY-related traits were measured, including KNPR, EL, EW and GW per plant.

**Table 3:** Pearson correlations among the studied traits and the heritability of these traits.

	KNPR	EL (mm)	EW (g)	GW (g)	H_b_^2^ (%)
KNPR	1.00				91.1
EL (mm)	0.83***	1.00			90.8
EW (g)	0.81***	0.78***	1.00		83.8
GW (g)	0.83***	0.77***	0.94***	1.00	83.0

KNPR, kernel number per row. EL, ear length. EW, ear weight. GW, grain weight.

*** indicates significance at p<0.001.

**Figure 3:**
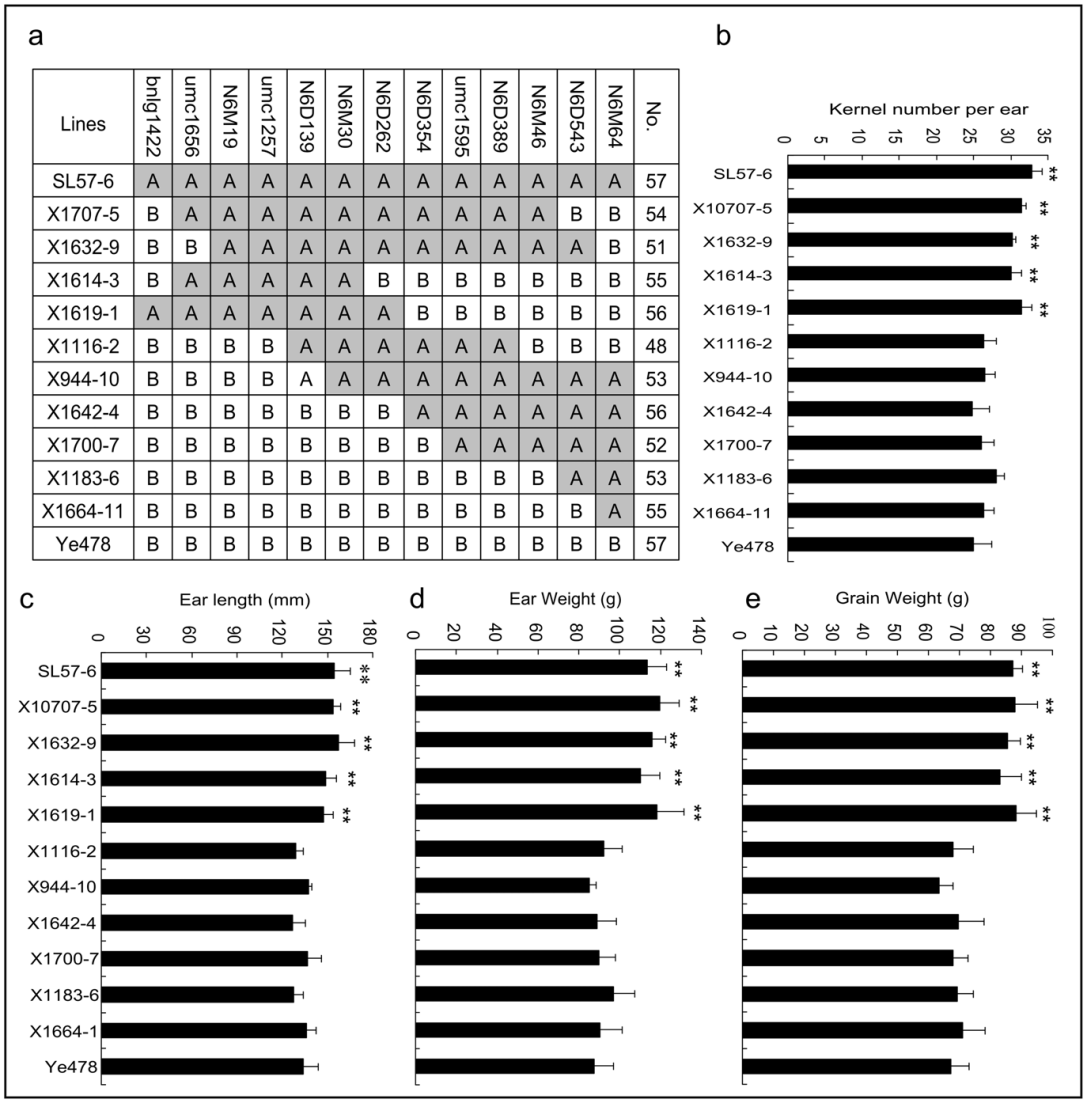
Genotype and phenotype of homozygous recombinant lines developed from a large F_2_ population in summer 2011. 3a shows the genotype of the recombinant lines, No. shows the number of samples, A and B represent marker genotypes that are the same as SL57-6 and Ye478, respectively. 3b–3e show phenotypes of kernel number per row (3b), ear length (3c), ear weight (3d) and grain weight per ear (3e) detected in winter 2011 at Sanya, respectively. **: Significant difference at P = 0.01.

### Molecular Marker Development and Linkage Analysis

The DNA sequence flanked by *umc1656* and *umc1857* in the B73 genome [26] was retrieved to develop SSR markers using the Simple Sequence Repeat Identification Tool [27], with the maximum motif-length set at four base pairs, and the minimum number of repeats set at 10 base pairs. Primers were designed using Primer Premier 5.0 with a product size under 300 bp. Similarly, PCR-based markers were developed in the chromosomal interval flanked by *N6M19* and *N6M66*. Those developed SSRs from the *Umc1656*-*Umc1857* interval were used to identify the genotypes of the F_2_ population in summer 2009, and to construct a linkage map. The PCR-based markers within *N6M19-N6M66* interval were used to identify the genotypes of the large F_2_ population in summer 2011. All of the developed markers that were used to screen the F_2_ population for recombinant chromosomes are listed in Table 1.

**Table 4:** QTLs detected in the F_2∶3_ families in summer 2010.

Population	Trait	Marker Interval	LOD	PVE (%)	Add	Dom
2010 F_2∶3_ families	KNPR	N6M19-N6M30	33.28	56.14	3.01	0.19
	EL (mm)	N6M19-N6M30	32.08	44.99	11.81	5.44
	EW (g)	umc1656-N6M19	23.55	41.76	12.36	2.99
	GW (g)	umc1656-N6M19	22.44	40.24	11.22	2.72
2011 F_2_	KNPR	N6M19-umc1257	69.95	23.34	2.01	0.93
	EL (mm)	N6M19-umc1257	20.43	7.48	5.18	0.67
	EW (g)	N6M19-umc1257	30.41	11.04	7.28	1.78
	GW (g)	N6M19-umc1257	26.60	9.72	6.42	−0.53

KNPR, kernel number per row. EL, ear length. EW, ear weight. GW, grain weight.

LOD, logarithms of odds.

Add, Additive. Dom, Dominant.

PVP (%), Percentage of phenotypic variance explained.

**Table 5:** Genotype and phenotype of homozygous recombinants selected from the F_2_ in 2011.

NIL	bnlg1422	umc1656	N6M19	umc1257	N6D139	N6M30	N6D262	N6D354	umc1595	N6D389	N6M46	N6D543	N6M64	No.	Mean±SD	P Value
SL57-6	A	A	A	A	A	A	A	A	A	A	A	A	A	30	32.9±2.5	1.19E-06
Rec1	B	B	A	A	A	A	A	A	A	A	A	A	A	3	29.2±3.3	2.76E-04
Rec2	A	A	A	A	A	A	A	A	A	A	A	A	B	39	30.0±2.4	9.42E-05
Rec3	B	B	A	A	A	A	A	A	A	A	A	B	B	4	30.7±2.1	6.07E-05
Rec4	B	A	A	A	A	A	A	A	B	B	B	B	B	2	30.1±2.7	3.78E-04
Rec5	A	A	A	A	A	A	A	B	B	B	B	B	B	14	27.9±1.8	8.25E-03
Rec6	A	A	A	A	A	B	B	B	B	B	B	B	B	7	29.3±2.5	1.19E-03
Rec7	A	A	A	A	B	B	B	B	B	B	B	B	B	4	29.3±1.5	2.21E-04
Rec8	A	A	B	B	B	A	A	A	A	A	A	A	A	5	23.3±3.8	0.67
Rec9	A	A	B	B	B	B	B	A	A	A	A	A	A	5	21.0±3.08	0.07
Rec10	B	B	B	B	B	B	B	B	A	A	A	A	A	11	22.4±2.39	0.06
Rec11	B	B	B	B	B	B	B	B	B	B	A	A	A	10	24.8±1.64	0.69
Rec12	B	B	B	B	B	B	B	B	B	B	B	A	A	67	23.2±3.24	0.09
Rec13	B	B	B	B	B	B	B	B	B	B	B	B	A	20	25.0±2.16	0.08
Ye478	B	B	B	B	B	B	B	B	B	B	B	B	B	30	24.4±1.40	

A and B represent marker genotypes that are the same as SL57-6 and Ye478, respectively.

No. shows the number of recombinants with the same genotype.

### QTL Analysis

The phenotypic variance among NILs was estimated by analysis of variance (ANOVA). Broad-sense heritability was estimated on a family mean basis. QTL analysis was first performed by ANOVA, testing the significance of the difference between phenotypic values of the genotypic classes (homozygous for the SL57-6 allele and homozygous for the Ye478 allele) at each marker position. A significance threshold of P = 0.01 was chosen for declaring linkage between a marker and *qKNPR6*. QTL mapping was performed using QTL IciMapping [28].

### Quantitative RT-PCR

Immature ears representing two different ear development stages (1 to 2 mm long) were collected from Ye478 and SL57-6, respectively. Total RNA was extracted using TRIzol® Reagents (Promega, Madison, WI, USA) according to the manufacturer’s instructions. Gene-specific primers (Table 1) were designed to assay the expression of six candidate genes by real-time RT-PCR. Total RNA was treated with RNase-free DNase (Promega) to remove contaminating DNA. DNA-free RNAs were mixed with 1.0 µg oligo dT on ice, and then heated to 70.0°C for 10 minutes, followed by three minutes on ice. First strand cDNA was synthesized using 1.0 µL M-MLV reverse transcriptase according to the manufacturer’s instructions (Takara, Otsu, Japan). cDNA (1.0 µL) was used as the template in PCR reactions, which contained 10.0 µL 2 × Smart SYBR QPCR Mix (DoGene, Shanghai, China), 0.8 µL 10 µM primer mixture and 8.2 µL water. Reactions were performed using the Chromo4 Real-Time PCR Detection System. The threshold value was empirically determined using the observed linear amplification phase of all primer sets. Sample cycle threshold (Ct) values were standardized for each template, based on an Actin (GRMZM2G030169) control reaction. The comparative Ct method (ΔΔCt) was used to determine the relative transcript abundance of each gene [29].

## Results

### qKNPR6 Mapping

Using 193 F_2_ plants developed by crossing SL57×Ye478 in 2009, *qKNPR6* was remapped to the *umc1656*-*umc1857* interval (24.6 cM) on 6.02–6.04 bin, explaining 39.45% of the phenotypic variance. No QTL was detected in the introgression segment on 4.08 bin (Figure 2). The allele of SL57 showed a positive additive effect and a negative dominant effect. Individuals with only one introgression segment on 6.02–6.04bin or on 4.08 bin were selected on the basis of marker genotype and were self-pollinated to produce two segmental isolines: SL57-6 harboring the *qKNPR6*, and SL57-4 with an introgressed segment on 4.08bin (Table 2). Comparing the phenotype among SL57-4, SL57-6 and Ye478, the KNPR was significantly greater in SL57-6 than in Ye478 across three environments (Table 2). However, the KNPR of SL57-4 was not significantly different from that of Ye478. The result agreed with the QTL mapping, and indicated that the introgressed segment within SL57-6 contains an allele that can increase KNPR relative to the Ye478 allele.

Furthermore, QTL mapping using 201 F_2∶3_ families provided more credible information on *qKNPR6*. ANOVA showed that variances of KNPR, EL, EW, and GW among families were significant at the *p<0.001* level. These traits showed high heritability, from 83% to 91%, and were highly positively correlated with each other. For example, coefficient correlations between KNPR and EL, EW and GW were higher than 0.8 (Table 3). Those developed markers in the umc1656-umc1857 interval were used to construct a dense linkage map and identify the QTL. Thus, *qKNPR6* was narrowed down to a 2.8cM interval flanked by *N6M19* and *N6M30*, and had a large additive effect (3.02 kernels), explaining 56.3% of the phenotypic variance. In the *N6M19*-*N6M30* interval, a QTL for EL was also detected that explained 44.99% of the phenotypic variance (Table 4 and Figure 2a). Moreover, two QTLs for EW and GW were identified in a nearby region.

The F_2_ individuals grown during the summer of 2009 were genotyped using markers within *umc1656-umc1857* and flanking markers to detect recombinant chromosomes in *qKNPR6*. Eight recombinants were self-pollinated to form sub-NILs, as shown in Figure 2b. Phenotypic evaluation indicated that sub-NILs harboring the introgressed segment flanked by *umc1656* and *N6M30* had higher KNPR than Ye478, while those lines with the Ye478 genotype in the corresponding interval, such as 627-3 and 633-1, did not significantly differ in terms of KNPR from Ye478 (Figure 2c). The result confirmed that the *umc1656-N6M30* interval contains a gene controlling KNPR in maize.

### Fine Mapping of *qKNPR6*

A larger segregating population with 12,800 F_2_ individuals was developed from the SL57-6×Ye478 cross and was used to finely map *qKNPR6*. Furthermore, newly developed markers were used to produce a high-resolution linkage map of the target QTL interval by genotyping 1,000 randomly selected plants. Using the genotype and phenotype of these random F_2_ samples, *qKNPR6* was further narrowed down to a 0.91 cM interval flanked by *N6M19* and *umc1257*. In the same interval, QTLs for EL, EW and GW were also detected (Table 3). The logarithms of odds (LOD) scores of these QTLs increased rapidly, up to 69.95 for KNPR. However, the phenotypic variance explained by the QTLs decreased, to only 23.34% by *qKNPR6*, and 7.48% by the EL QTL (Table 4). The unexpected decline could be attributed to the high environmental sensitivity of these GY-related traits. To decrease the environmental effect on the phenotype of these traits, 363 recombinant chromosomes were detected by genotyping all of F_2_ plants using markers within and flanking *qKNPR6*. Of these recombinant chromosomes, 191, representing 13 meiotic events, were open-pollinated to evaluate their phenotypes in summer 2011. The phenotypic means of recombinants with the same genotype were used to detect QTLs by a t-test. The result demonstrated that Rec1 to Rec7 carried the SL57-6 allele in the N6M19-umc1257 interval, while Rec8 to Rec13 carried the Ye478 allele in the corresponding interval. The KNPR of Rec1 to Rec7 was distinctly higher than that of Ye478, as well as being higher than that of Rec8 to Rec13 (Table 5), indicating that *N6M19*-*umc1257* interval might contain a QTL for KNPR. The remaining recombinants (172) were self-pollinated to develop homozygous recombinant lines (sub-NILs), and were then phenotypically assessed in winter 2011 at Sanya (Southern China). The result of QTL mapping using the sub-NILs was consistent with the results of linkage mapping in the F_2_ population and of the substitution mapping using the recombinants, supporting the view that *qKNPR6* is harbored within the *N6M19-umc1257* interval (Figure 3a,3b). Notably, those sub-NILs holding the favorable allele of *qKNPR6* also showed greater performance in EL, EW and GW per ear than Ye478 (Figure 3c–3e), indicating that the N6M19-umc1257 region might affect the performance of multiple traits, i.e., it could be a pleiotropic locus.

### Prediction of Candidates and Expression Assays

**Figure 4:**
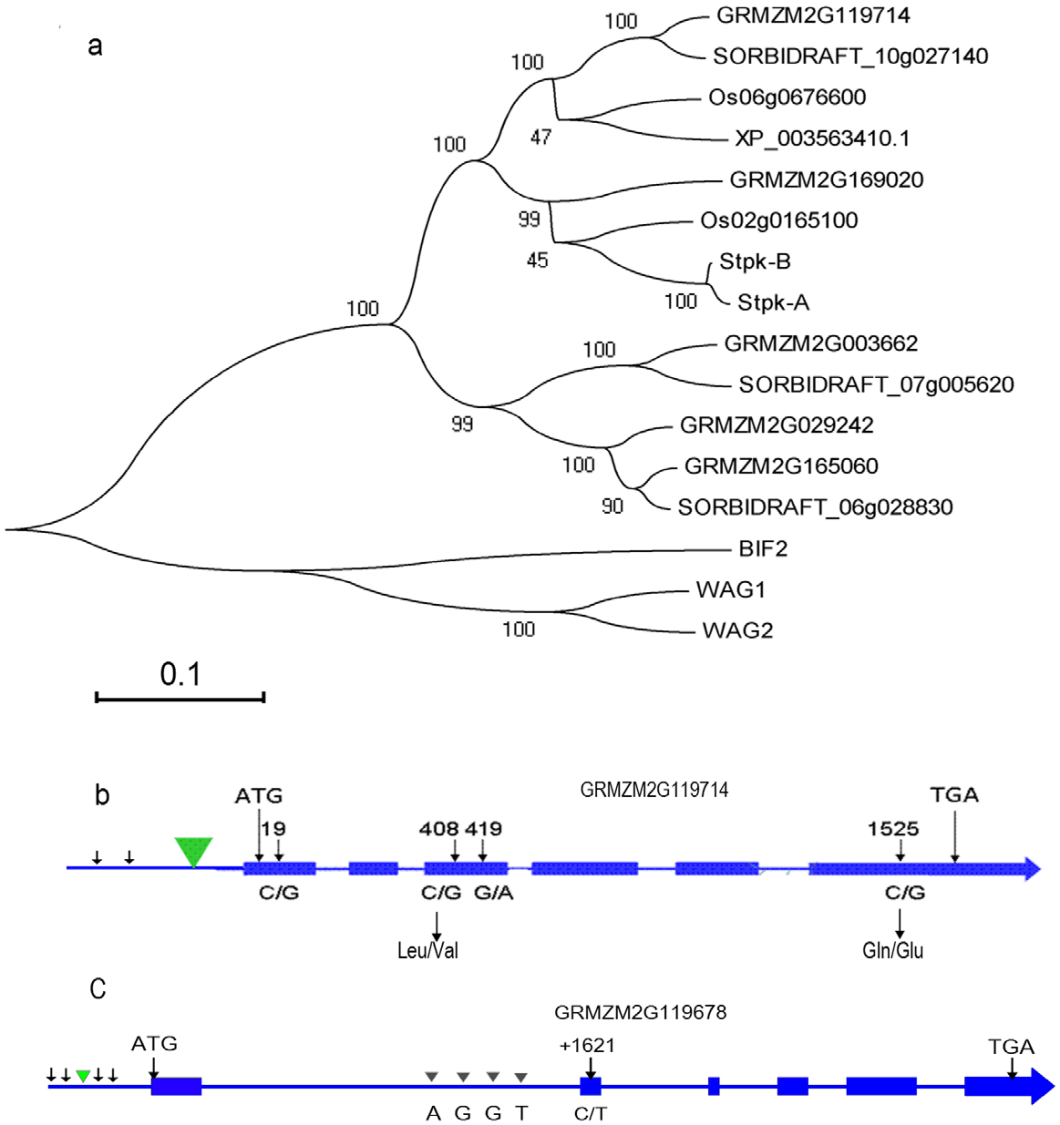
Diversity of serine/threonine protein kinase receptor gene and SET domain-containing protein gene. 4a: The phylogenetic tree of serine/threonine protein kinase receptor proteins from several plant species. GRMZM2G119714, which encodes a serine/threonine protein kinase receptor, is clustered into a different clade from Bif2, a known serine/threonine protein kinase in maize. 4b: The nucleotide and amino acid variation of serine/threonine protein kinase receptor protein between Ye478 and SL57-6. 4c: The nucleotide variation of SET domain-containing protein genes between Ye478 and SL57-6.

**Figure 5:**
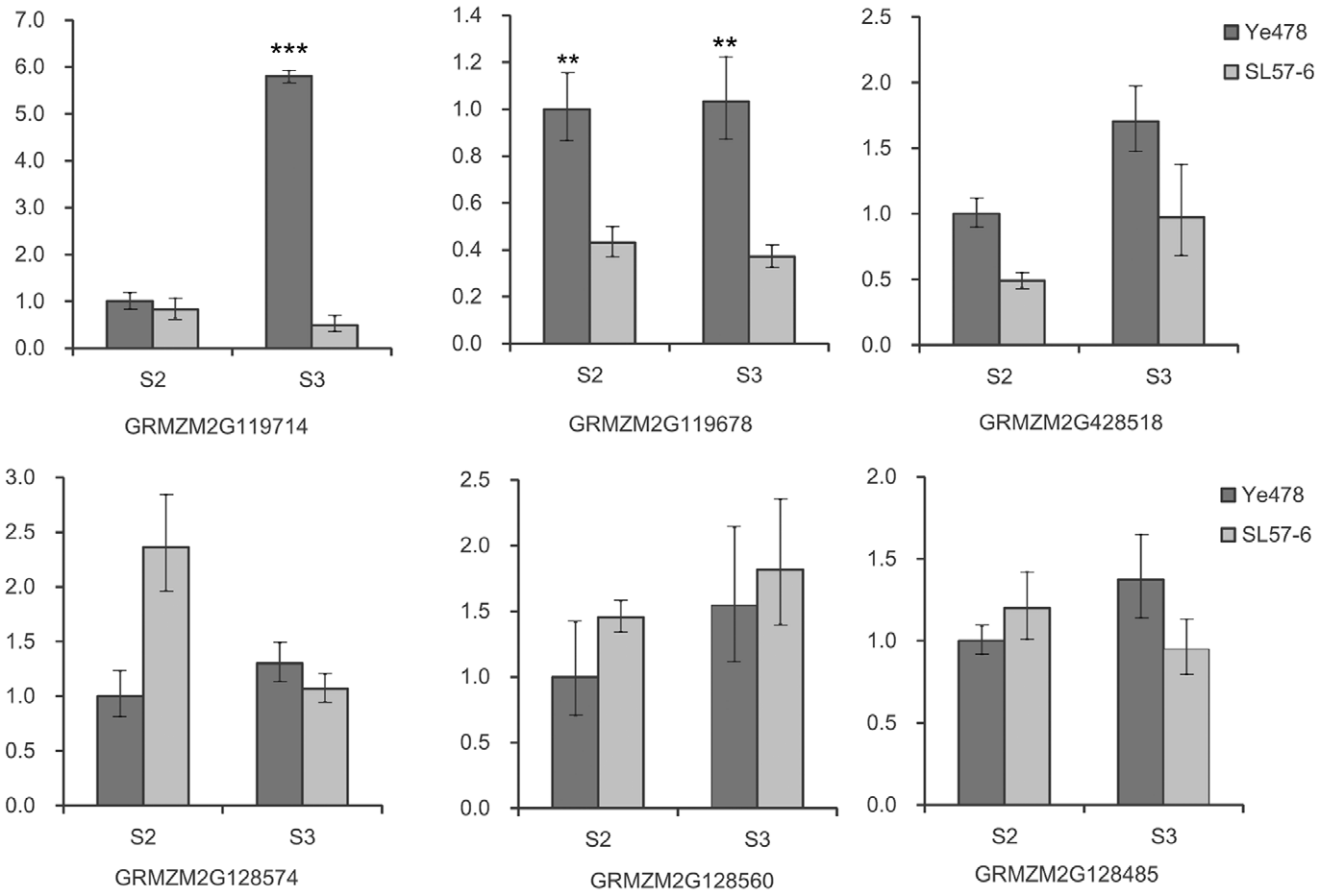
Relative level of candidate gene expression in immature ears. S2: Differentiation stage of the spikelet-pair meristem. S3: Differentiation stage of the spikelet meristem. **: Significant difference at P = 0.01, ***: Significant difference at P = 0.001.

The *N6M19-umc1257* interval on the B73 genome is about 198 Kb long, and contains six genes annotated in B73 RefGen_v2. GRMZM2G119714 encodes a serine/threonine protein kinase receptor (STKR) protein that is highly homologous with receptor protein kinase PERK-like in *Oryza sativa*, but markedly differs from Bif2 of maize (Figure 4a). GRMZM2G119678 encodes a SET domain-containing protein group 102 (SDG102), which is highly homologous with *Arabidopsis* ASHH2/SDG8 [30]. GRMZM2G428518 encodes a protein homologous with alpha-glucosidase in *Oryza sativa*, and GRMZM2G128485 encodes a BTB superfamily protein that is 61% homologous with a *Brachypodium distachyon* BTB/POZ domain-containing protein that is involved in auxin signaling. The other two genes are of unknown function. A qPCR assay revealed that the six genes were expressed in the immature ears of both SL57-6 and Ye478. The expression of *STKR* in Ye478 at the spikelet meristems (SMs) stage was significantly higher than in SL57-6. The expression of *SDG102* in Ye478 was markedly higher than in SL57-6 at SPM and SM developmental stages (Figure 5). The expressions of the other candidates showed no significant differences between Ye478 and SL57-6 at the p = 0.01 level. Sequencing demonstrated that the coding regions of both *STKR* and *SDG102* were highly conserved in Ye478 and SL57-6. Four SNPs were identified in the coding region of *STKR,* of which two C/G SNPs led to amino acid substitutions: Leu to Var and Gln to Glu (Figure 4b). The two amino acids are not conserved among plant species. The 5′-upstream sequence of *SDG102* has a few SNPs between Ye478 and SL57-6, and only one C/T SNP was found in the coding region of *SDG10*2 (Figure 4c). However, the promoter region of the *STKR* gene showed a large insertion/deletion variation (Figure 4b), which might affect the expression level of the gene in the two NILs.

**Figure 6:**
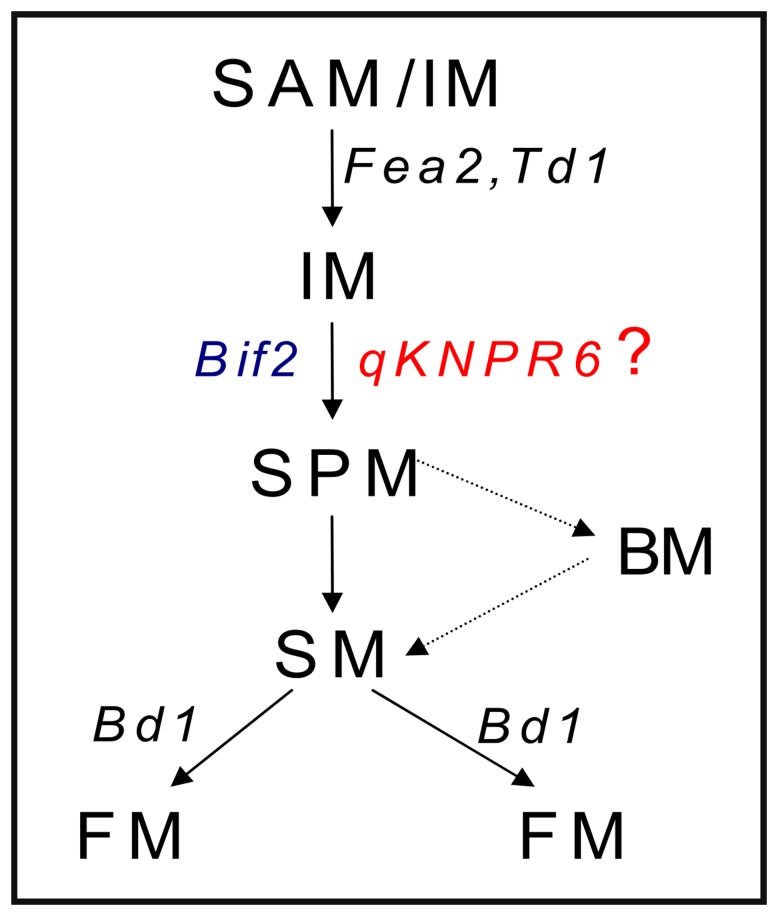
Schematic diagram of inflorescence development along with genes that are likely to affect the various developmental stages. SAM, shoot apical meristem; AM, axillary meristem; IM, inflorescence meristem; SPM, spikelet-pair meristem; SM, spikelet meristem; FM, floret meristem. The genes were: *Fea2*, *fasciated ear2*; *Td1*, *thick tassel dwarf1*; *Bif2*, *barren inflorescence2*; *Bd1*, *branched silkless1*; *qKNPR6*, a QTL for kernel number per row on chromosome 6.

## Discussion

### The qKNPR6 Might be a Pleiotropic Locus

Most agronomically important traits in crop plants, such as GY, quality, and resistance to biotic and abiotic stresses, are complex quantitative traits that are usually controlled by multiple QTLs which are frequently clustered on the genome [1]–[31], [32]–[33], [34]–[35]. The clustered QTLs can be genetically explained by QTL pleiotropy or tight linkage of QTLs. QTL analysis typically produces a large confidence interval spanning 10–30 cM, comprising several hundred genes; therefore, it is usually uncertain whether a QTL represents one or multiple genes, which makes it difficult to ascertain whether a QTL cluster results from a pleiotropic gene or from multiple linked genes. In one case, clustered QTLs were resolved in linked QTLs with minor effects. QTL mapping studies in maize clearly showed that clustered QTLs for complex traits, such as plant height and GY, could be separated into multiple QTLs when the resolution of the linkage analysis improved from a 10–30 cM to a 1–5 cM interval [36]–[37], [38]–[39]. In another case, a QTL cluster was mapped as a major QTL affecting multiple traits that correlated with each other: the QTL acts as a regulator in multiple biological pathways [35]–[40], [41]–[42]. Several cloned QTLs have provided evidence of gene pleiotropy. For example, *vgt1* controls flowering time and plant node number in maize [40], *tb1* affects the plant and inflorescence architecture [43], and *Ghd7* and *Ghd*8 simultaneously affect GY, plant height and heading date in rice [41]–[42].

In the present study, KNPR in maize was significantly correlated with EL, as well as with EW and GW (Table 3). A developmental association of the two traits could explain the highly positive correlation between KNPR and EL. The correlation between KNPR and GW could be attributed to the indirect effect of alteration of KN on EW per plant. QTL mapping in F_2∶3_ families indicated that multiple QTLs for the above-mentioned traits were clustered in a ∼2.8 cM interval (Table 4). When the clustered QTLs were further narrowed down to an approximately 198Kb region by substitution mapping, the QTL’s simultaneous effects on the performance of the four association traits were still observed (Figure 3). This suggested that *qKNPR6* might be a pleiotropic locus that simultaneously affects KNPR and EL, and indirectly affects EW and GW per plant in maize.

### Possible Candidate Gene Underlying the qKNPR6

Maize female inflorescence originates from an axillary meristem at the tip of a lateral shoot in the axil of a leaf. When the plant transitions from vegetative to reproductive development, the axial meristem becomes an IM, and the IM produces multiple rows of SPMs, a kind of short BM, which then form two SMs. Each SM uniquely initiates two floral meristems (FMs) (upper FM and lower FM), and each FM subsequently forms the floral organs. Subsequently, the lower floret and the stamens abort, resulting in the formation of single female florets. Obviously, a kernel in maize ear is developed from a pollinated floret. This implies that the number of fertile florets in a given row of SMs determine the possible KN in the row. In other words, maintaining a high differentiating activity in the SM is important for developing more FMs that produce fertile florets. Thus, those genes that are involved in regulation of the differentiating capacity of IMs are probable candidates for genetic control of KNPR in maize. Within the 198Kb region of *qKNPR6*, the B73 genome encodes six genes with EST evidence and all six genes are expressed in maize immature ears [44].

The expression level, sequence variation and the annotated biological function in model species of the six genes in SL57-6 and Ye478 could provide clues to identity the candidate gene. Among the six predicted candidates for *qKNPR6*, quantitative PCR revealed that the *STKR* and *SDG102* were differentially expressed in two NILs, Ye478 and SL57-6, while the other genes displayed similar expression levels in the two NILs and had similar expression patterns during the different developmental stages of the immature ear (Figure 5). Temporal and spatial expression patterns showed that the serine/threonine-protein kinase receptor gene is highly expressed in the immature ear, roots, 20-day endosperm, ovule, and tassel, but is not expressed in pollen or leaves [44]. Previous studies demonstrated that *Barren inflorescence2* (*Bif2*) encodes a serine/threonine protein kinase [12] that phosphorylates ZmPIN1a to regulate the subcellular localization of ZmPIN1a [45]. *Bif2* mutants produce rudimentary ears that occasionally produce spikelets [13]. In the 198Kb segment, one candidate gene encodes a receptor protein serine/threonine kinase (STKR) that plays a role in the regulation of cell proliferation, cell differentiation, and embryonic development by phosphorylating receptor proteins. Sequencing revealed that the coding regions of the *STKR* gene was highly conserved, while the promoter region showed a large insertion/deletion variation, which might affect the expression level of the gene in Ye478 and SL57-6. Another differentially expressed gene, *SDG102*, is a homolog of Arabidopsis *ASHH2/SDG8* [30], which is a class of proteins that have been implicated in regulating gene expression through H3K36 trimethylation modification [46]. *ASHH2/SDG8* is a repressor of the transition from vegetative to reproductive growth [47]–[48], and is a regulator of shoot branching, flower morphology and fertility in *Arabidopsis* [49]. *ASHH2/SDG8* mutants can downregulate expression of the floral organ identifying genes *APETALA1* (*AP1*) and *AP3*, display homeotic changes of floral organs and have very low seed sets because of developmental defects of reproductive organs. By inference, we suggest that one of the two genes is a likely candidate for *qKNPR6*, owing to their function in inflorescence development and floral organ differentiation, but direct evidence from map-based cloning, genetic transformation and mutants are still required.

If one of the two genes is responsible for the *qKNPR6*, how does it act to control KNPR in maize? Upadyayula et al. (2006) summarized the genetic steps of the functions of several cloned genes in the development of the tassel and ear (Figure 6) [50]. Both *Fasciated ear2* (*Fea2*) and *Thick tassel dwarf1* (*td1*) have similar functions in the transition from AM or SAM to IM. *Barren inflorescence2* (*Bif2*) affects the transition from IM to SPM or BM, and both *Ra1* and *Ra2* have similar functions in the transition from SPM to BM. *Branched silkless1* (*Bd1*) is required for FM identity, and regulates the transition from SM to FM. Notably, *Bif2*, which encodes a serine/threonine protein kinase co-orthologous to PINOID [12], is required for maintenance of the BM, SM and FM in the inflorescence. During inflorescence development, BIF2 directly phosphorylates ZmPIN1a, and regulates auxin transport by regulation of the subcellular localization of ZmPIN1a [45]. In addition, the heterochronic expression of a gene is considered a regulator of phenotypic variation of a quantitative trait, such as *fw2.2* [51] and *Ghd7* [41]. Thus, we postulate that the STKR protein might function together with BIF2 to positively regulate auxin efflux by phosphorylation of PIN1a. The heterochronic expression of *qKNPR6* in an inflorescence might regulate auxin concentration and its temporal and spatial distribution, affecting the activity duration of the BM, SM and FM, which in turn would regulate the KN in a maize ear. If the *SDG102* is responsible for the *qKNPR6*, on the basis of function of its homolog of Arabidopsis *ASHH2/SDG8*, then *qKNPR6* is postulated to be a repressor of transition from vegetative to reproductive growth. More FMs (or kernels) in female inflorescence of SL57 than that of Ye478 might be explained by lower expression of *SDG102* in SL57 inflorescence allowing earlier transition from vegetative to reproductive growth.
